# Objective assessment of tumor infiltrating lymphocytes as a prognostic marker in melanoma using machine learning algorithms

**DOI:** 10.1016/j.ebiom.2022.104143

**Published:** 2022-07-07

**Authors:** Thazin Nwe Aung, Saba Shafi, James S. Wilmott, Saeed Nourmohammadi, Ioannis Vathiotis, Niki Gavrielatou, Aileen Fernandez, Vesal Yaghoobi, Tobias Sinnberg, Teresa Amaral, Kristian Ikenberg, Kiarash Khosrotehrani, Iman Osman, Balazs Acs, Yalai Bai, Sandra Martinez-Morilla, Myrto Moutafi, John F. Thompson, Richard A. Scolyer, David L. Rimm

**Affiliations:** a Department of Pathology, Yale School of Medicine, New Haven, CT, USA; b Melanoma Institute Australia, The University of Sydney, Sydney, NSW, Australia; c Adelaide Medical School, The University of Adelaide, Adelaide, SA, Australia; d Department of Oncology and Pathology, Karolinska Institutet, Stockholm, Sweden; e Department of Clinical Pathology and Cytology, Karolinska University Laboratory, Stockholm, Sweden; f University Tübingen, Tübingen, Germany; g University of Queensland, UQ Diamantina Institute, Brisbane, QLD, Australia; h Department of Medicine, Grossman School of Medicine, New York University, USA; i Department of Internal Medicine (Medical Oncology), Yale University School of Medicine, New Haven, CT, USA; j Faculty of Medicine and Health, The University of Sydney, Sydney, NSW, Australia; k Tissue Pathology and Diagnostic Oncology, Royal Prince Alfred Hospital and NSW Health Pathology, Sydney, NSW, Australia; l Charles Perkins Centre, The University of Sydney, Sydney, NSW, Australia; m Cluster of Excellence iFIT (EXC 2180) “Image-Guided and Functionally Instructed Tumor Therapies”, 72076 Tübingen, Germany; n Institute of Pathology and Molecular Pathology, University Hospital Zurich, Zurich, Switzerland; o Department of Dermatology, Princess Alexandra Hospital, Brisbane, QLD, Australia

**Keywords:** Tumor-infiltrating lymphocytes (TILs), Digital image analysis, Machine learning cell segmentation algorithm, Early-stage melanoma, Prognostic marker

## Abstract

**Background:**

The prognostic value of tumor-infiltrating lymphocytes (TILs) assessed by machine learning algorithms in melanoma patients has been previously demonstrated but has not been widely adopted in the clinic. We evaluated the prognostic value of objective automated electronic TILs (eTILs) quantification to define a subset of melanoma patients with a low risk of relapse after surgical treatment.

**Methods:**

We analyzed data for 785 patients from 5 independent cohorts from multiple institutions to validate our previous finding that automated TIL score is prognostic in clinically-localized primary melanoma patients. Using serial tissue sections of the Yale TMA-76 melanoma cohort, both immunofluorescence and Hematoxylin-and-Eosin (H&E) staining were performed to understand the molecular characteristics of each TIL phenotype and their associations with survival outcomes.

**Findings:**

Five previously-described TIL variables were each significantly associated with overall survival (*p*<0.0001). Assessing the receiver operating characteristic (ROC) curves by comparing the clinical impact of two models suggests that etTILs (electronic total TILs) (AUC: 0.793, specificity: 0.627, sensitivity: 0.938) outperformed eTILs (AUC: 0.77, specificity: 0.51, sensitivity: 0.938). We also found that the specific molecular subtype of cells representing TILs includes predominantly cells that are CD3+ and CD8+ or CD4+ T cells.

**Interpretation:**

eTIL% and etTILs scores are robust prognostic markers in patients with primary melanoma and may identify a subgroup of stage II patients at high risk of recurrence who may benefit from adjuvant therapy. We also show the molecular correlates behind these scores. Our data support the need for prospective testing of this algorithm in a clinical trial.

**Funding:**

This work was also supported by a sponsored research agreements from Navigate Biopharma and NextCure and by grants from the NIH including the Yale SPORE in in Skin Cancer, P50 CA121974, the Yale SPORE in Lung Cancer, P50 CA196530, NYU SPORE in Skin Cancer P50CA225450 and the Yale Cancer Center Support Grant, P30CA016359.

**Research in context**

**Evidence before this study**

TIL scores visually assessed by the pathologists have not been broadly clinically implemented due to the lack of reproducibility caused by subjective assessment between pathologists and institutions. Automated TIL% (eTILs%) score, defined by the machine learning algorithm NN192, developed using open-source software, QuPath, has been shown to be prognostic in melanoma^1^ but its clinical utility has not yet been broadly proven.

**Added value of this study**

This study pools patients with melanoma from a series of international cohorts and supports the previous finding that automated TIL score (eTIL%) is an independent prognostic marker in primary melanoma patients.

**Implications of all the available evidence**

we additionally show that the prognostic performance of eTIL% is stage specific. The use of NN192 machine learning algorithm could be a valuable and easy-to-implement tool for prospective testing of patients with early-stage melanoma and could be validated as a selector for patients that can safely omit immunotherapy in the adjuvant setting.

Alt-text: Unlabelled box

## Introduction

Melanoma is considered a highly immunogenic tumor and is responsive to immunotherapy.^2^ Checkpoint inhibitor immunotherapies targeting programmed cell death protein 1 (PD-1) or cytotoxic T-lymphocyte-associated protein 4 (CTLA-4) have been shown to significantly improve the overall survival of patients with advanced stage metastatic disease.^3^^,^^4^ Furthermore, adjuvant treatment with immune checkpoint blockade (or BRAF pathway targeted therapy in BRAF mutant melanoma patients) improved relapse-free survival (RFS) compared to placebo in phase 3 clinical trials^5^^,^^6^ and is now regarded as standard of care in high-risk stage III melanoma patients. Furthermore, it was recently reported that adjuvant anti-PD1 immunotherapy improved relapse-free survival in high-risk stage II melanoma patients. However, up to 30% of stage III patients treated with adjuvant immunotherapy developed disease recurrence. Furthermore, treatment- related adverse events occur in at least one in five patients, and treatment related fatalities have been reported in up to one in one hundred patients.^5^^,^^7^ There is therefore an urgent need to identify patients at high risk of disease relapse who may benefit from adjuvant therapies and those patients at low risk of relapse who can be safely spared further treatment and the concomitant risks of treatment related adverse events.

Tumor-infiltrating lymphocytes (TILs) reflect the host's immune response against cancer cells.^2^^,^^8^ Correlation between different TILs infiltrates and improved survival in melanoma patients has been reported in multiple studies.^9^, ^10^, ^11^, ^12^ Traditionally, TIL scores have been visually assessed by the pathologists, but due to the lack of reproducibility caused by subjective assessment between pathologists and institutions, TIL scoring techniques have not been broadly clinically implemented.^13^ To increase objectivity, a machine learning algorithm developed using open source software, QuPath, has been shown to facilitate the investigation of complex spatial patterns by firstly classifying four cell types, including tumor cells, TILs, stromal cells, and “other” cells, for the assessment of the proportion TILs within different cell populations.^1^^,^^14^ Acs et al. highlighted the robustness of the NN192 machine learning algorithm by comparing the performance between eTIL scores and pathologist TIL scores.^1^ The latter study also showed that TILs score assessed by the NN192 algorithm was an independent prognostic marker in melanoma.

Here, we used the same cell classifier to validate the association of percent electronic TILs (eTIL%) with disease-specific survival in patients with melanoma in a broad set of cohorts from melanoma centers around the world. For this effort, we used the previously established cut-point of eTIL%^1^ to test The Cancer Genome Atlas (TCGA) melanoma cohort and four other melanoma cohorts from Yale School of Medicine, Melanoma Institute Australia, Tubingen University, and New York University. Furthermore, we also tested both cell types and area, variables that pathologists cannot easily calculate, for their prognostic significance. The goal of this effort is not to help pathologists more accurately assess TIL, but to validate this prognostic assay for potential pathologist-independent use in future prospective trials to determine which melanoma patients might be spared immunotherapy in the adjuvant setting. We aimed to specify the most reliable operator-independent identification of eTIL%, which can be prospectively validated in future studies. Finally, we assessed the prognostic role of the immunophenotypic subtypes of TILs, as defined by CD45+, CD3+, CD4+, CD8+, CD56+, CD20+, and FOXP3+ cells, using the Yale cohort to better understand the TIL subsets/phenotypes and their associations with patients' survival. Our ultimate goal was to determine the best approach for utilizing TIL infiltrates to predict disease outcome in melanoma patients that can be validated prospectively with the goal of proving clinical utility to select the high-risk subset of melanoma patients that are likely to not require immunotherapy in the adjuvant setting.

## Methods

### Study population

We assessed retrospectively collected samples from 5 independent cohorts from: (1) the Department of Pathology, Yale University, (2) the Melanoma Institute Australia (MIA), (3) Tubingen University, (4) TCGA and (5) Langone Medical Center, New York University (NYU). Two cohorts were in tissue micro-array (TMA) format and 3 in Whole Tissue Sections (WTS) format. The Yale University cohort comprised tissues from 187 stage I and II patients diagnosed between 1998 and 2011 with a median follow-up of 64 months. The Tubingen University TMA cohort consisted of 231 stage I and II and 20 stage III and IV melanoma patients diagnosed between 1992 and 2000 with 97 months median follow-up. The MIA WTS cohort consisted of 55 stage I and II patients and 41 stage III and IV patients diagnosed between 1998 and 2019 with 70.3 months median follow-up. The NYU WTS cohort consists of 88 high-staged patients diagnosed between 2009 and 2019 with 2.3 months median follow-up. The publicly available WTS-TCGA melanoma cohort comprised 139 stage I and II patients diagnosed between 1994 and 2013 with 38.6 months median follow-up (https://portal.gdc.cancer.gov/repository). Patients in the TCGA cohort were classified according to the 4^th^, 5^th^, 6^th^, and 7^th^ Editions of the American Joint Committee on Cancer (AJCC) tumor, node, metastasis (TNM) staging system. All other cohorts were classified according to the 8^th^ edition of the AJCC tumor,^15^^,^^16^ node, metastasis (TNM) staging system (Table 1).

**Table 1:** Clinicopathological features of five cohorts from multiple institutions.

Characteristic	Sydney Cohort (n=97)	Tubingen Cohort (n=253)	Yale Cohort (n=202)	NYU Cohort (n=88)	TCGA Cohort (n=139)
	N (%)	N (%)	N (%)	N (%)	N (%)
Age	NA	NA			
Median (Range)			63 (6-98)	69.7 (24.5-89.5)	63 (15-90)
Gender	NA	NA			
Male			116 (60.7)	54 (61.4)	53 (38.1)
Female			76 (39.3)	34 (38.6)	86 (61.9)
Morphology			NA	NA	NA
Superficial spreading	21 (21.6)	200 (79.1)			
Lentigo maligna	3 (3.2)	20 (7.9)			
Nodular	40 (41.2)	17 (6.7)			
Other	33 (34.0)	14 (5.5)			
Breslow depth					
Median (Range)	3.30 (1.05-50.00)	0.70 (0.13-20.00)	1.40 (0.50-16.00)		
Ulceration					
Yes	42 (43.3)	23 (9.1)	37 (19.4)		
No	46 (47.4)	228 (90.1)	154 (80.6)		
Stage (8^th^ Edition)					Stage (4th, 5th, 6th, 7^th^ Edition)
I	0 (0)	202 (79.8)	124 (66.3)	0 (0)	41 (29.5)
II	56 (57.7)	25 (9.9)	63 (33.7)	0 (0)	98 (70.5)
III	41 (42.3)	17 (6.7)	0 (0)	27 (30.7)	0 (0)
IV	0 (0)	7 (2.8)	0 (0)	61 (69.3)	0 (0)
Clark's level		NA		NA	NA
I	0 (0)		1 (0.6)		
II	0 (0)		2 (1.1)		
III	11 (11.3)		30 (16.6)		
IV	62 (63.9)		144 (79.6)		
V	22 (22.7)		4 (2.2)		
BRAF status		NA	NA		NA
Wild type	0 (0)			61 (69.3)	
Mutant	0 (0)			22 (25)	
Not Assessed	72 (74.2)			5 (5.7)	
BRAF positive	8 (8.2)			0 (0)	
NRAS positive	3 (3.1)			0 (0)	
Both Negative	14 (14.4)			0 (0)	
Treatment	NA	NA	NA		NA
Ipilimumab (Ipi)				8 (9.1)	
Nivolumab (Nivo)				6 (6.8)	
Ipi+Nivo				16 (18.2)	
Ipi/(Ipi/Nivo)				20 (22.7)	
Pembrolizumab				30 (34.1)	
Other treatment				8 (9.1)	
Dead of disease					NA
Yes	38 (39.2)	28 (11.1)	54 (26.3)	28 (31.8)	
No	59 (60.8)	225 (88.9)	148 (73.7)	60 (68.2)	
Dead of any cause			NA	NA	
Yes	46 (47.4)	49 (19.4)			26 (18.7)
No	51 (52.6)	204 (80.6)			113 (81.3)

### Ethics

All tissue samples from Yale cohorts were collected with approval from the Yale Human Investigation Committee protocol #9505008219. Written informed consent, or waiver of consent in some cases, was obtained from Yale cohort patients with the approval of the Yale Human Investigation Committee. Tissue samples from MIA cohort were collected with approval from the Sydney Local Health District (RPAH Zone) protocols #X17-0312 & 2019/ETH07604 and #X15-0311 & 2019/ETH06854. Tissue samples from Tuebingen cohort were collected with approval protocol #883/2019BO2. Tissue samples from NYU cohort were collected from the NYU Interdisciplinary Melanoma Cooperative Group: A Clinicopathological Database protocol # C10362. To ensure scientific integrity, the investigator was blinded to the clinical information during image processing.

### Digital image analysis using NN192 algorithm

H & E images for the Yale cohort TMA, and the WTS H & E slides from the Sydney and NYU cohorts were digitised using the Aperio ScanScope XT platform (Leica Biosystems, Wetzlar, Germany) slide scanner at 20x with a pixel size of 0.4986 µm x 0.4986 µm. The WTS digital TCGA images were downloaded from the NIH CDC porta specimen repository (https://portal.gdc.cancer.gov/repository). The H & E-stained TMA slide of the Tubingen cohort was digitised using Hamamatsu Nanozoomer HT slide scanner at 20x with a pixel size of 0.4986 µm x 0.4986 µm. QuPath open-source software (version 0.1.2)^7^ based NN192 melanoma machine learning algorithm with neural network method^1^ was applied for cell classification in this study. For WTS, the tumor and a 1–2-millimeter (mm) diameter surrounding tumor microenvironment to be analysed were carefully selected for accurate prediction of TILs. The area selection was reviewed by a pathologist. Due to the varying intensity both between and within cohorts, the “estimate stain vectors”, ESV, function in QuPath was used to refine the H&E stain for each digitised slide. The workflow for stain normalisation using ESV function was shown in Supplementary Figure 2. The number of cells identified as tumor and immune cells (in %) across multiple centers were shown in Supplementary Figure 3. Cell segmentation and classification were performed using the parameters previously described.^1^

### Assessment of eTILs using five variables

The machine-defined TILs variables were constructed using five different methods, as previously described.^17^ The first and established method was to calculate eTIL% representing the proportion of TILs over tumor cells, calculated as (TILs/TILs + tumor cells) x 100.^1^ Four additional methods were used to measure TILs as follows:

1) Measurement of the proportion of TILs over total cells: etTIL % = (TILs/total cells) x 100
2) Measurement of the proportion of TILs over stromal cells: esTIL % = (TILs/total cells – tumor cells) × 100
3) Measurement of the density of TILs over tumor region: eaTILs (mm^2^) = TILs/sum of tumor region areas analysed (mm^2^)
4) Measurement of the density of TILs over stromal area: easTIL % = [sum of TIL area (mm^2^) /stroma area (sum of tumor region areas analysed (mm^2^) – sum of tumor cell area (mm^2^))] × 100

The last method mimics the manual pathologist scoring of stromal TILs according to the International Immuno-Oncology Biomarker Working Group on Breast Cancer.^18^ The variables are shown schematically in Figure 3a.

### Immunofluorescence staining for immunophenotypic subtyping of TILs

Eleven commercially available antibodies including CD34 (1:4500; clone: QBE10, Dako), CD56 (1:200; clone: 123C3, Cell Signaling Technology), CD66b (1:500; clone: 80H3, LifeSpan Biosciences), FOXP3 (1:100; clone: D2W8E, CST), CD8 (1:250; clone: 144B, Dako), CD14 (1:500; clone: D7A2T, Cell Signaling Technology), CD3 (1:100; clone: SP7, Novus Biologicals), CD45 (1:200; clone: 2B11 + PD7/26, Dako), CD68 (1:200; clone: C8/144B, Dako), CD20 (1:150; clone: L26, Dako) and CD4 (1:100; clone: SP35, SpringBio) were tested in four multiplex panels including: (1) CD14/CD66b/CD68/S100/DAPI, (2) CD14/CD45, CD34/S100/DAPI, (3) CD3/CD56/CD20/S100/DAPI and (4) CD4/CD8/FOXP3/S100/DAPI. Multiplexed immunofluorescent (IF) staining on four serial sections of Yale TMA 76 (YTMA-76) was performed as described previously for simultaneous detection of multiple markers.^19^ Briefly, formalin-fixed paraffin-embedded (FFPE) TMA sections were deparaffinised and incubated using xylene and ethanol. The pretreatment heating device PT Module (Lab Vision, Thermo Fisher Scientific) was used for antigen retrieval in EDTA buffer pH 8 at 97°C for 20 minutes. To block endogenous peroxidase activity, 2.5% hydroxyl peroxide in methanol was used, and incubated the TMA slides for 30 minutes at room temperature. Non-specific antigens were then blocked with 0.3% Bovine Serum Albumin in 0.1 mol/L of Tris-buffered saline with 0.05% Tween 20 for 30 minutes. TMA sections were then incubated with the primary antibodies of interest. Primary monoclonal antibodies for cell profiling were co-incubated or sequentially incubated one after the other at room temperature for 1 hour, followed by the incubation of three horseradish peroxidase (HRP)-conjugated secondary antibodies at room temperature for 1 hour before tyramide-based labeling for 10 min. To quench HRP activity, the sections were incubated with 1 mM benzoic hydrazide solution with 0.15% hydrogen peroxide for 10 minutes. The secondary antibodies used in this study were anti-rabbit EnVision (Dako), anti-mouse EnVision (Dako), anti-mouse IgG3 (1:700; Abcam), and anti-mouse IgG2a (1:200; Abcam). The substrates were biotin tyramide (1:50; PerkinElmer), TSA Plus Cy3 tyramide (1:100; PerkinElmer), and Cy5 tyramide (1:50; PerkinElmer), respectively. Sections were then treated with streptavidin–Alexa Fluor 750 conjugate (1:100; Invitrogen) for 1 hour. Finally, to identify melanoma cells, sections were incubated with mouse anti-S100 (1:100; 15E2E2; BioGenex) and goat anti-mouse Alexa 488 (1:100; Invitrogen) for 1 hour. The slides were then counterstained with 4′,6-diamidino-2-phenylindole (DAPI) and mounted with ProLong Gold Mounting Medium (Invitrogen) to visualise nuclei.

### Multispectral image acquisition and cell counting on serial sections YTMA-76

Image acquisition of the stained slides was performed using Vectra/Polaris (Akoya Biosciences, Marlborough, MA) microscope to obtain MSIs (multispectral images). Briefly, a low magnification scan of the whole TMA slide was performed at 4×. The regions of interest (ROI) scan were then selected from a low-resolution using the Phenochart viewer (Akoya Biosciences), and the ROIs were subsequently acquired at the higher resolution MSIs at 20×. To analyse the MSIs, the spectra were extracted from acquired images to build the spectral library consisting of all fluorophores using inForm image analysis software version 2.4.9 (Akoya Biosciences), and the absence of spectral overlap between channels was checked by evaluating the unmixed images. The acquired multispectral images were then decomposed using a spectral library. Tumor, stroma, and background were identified using the trainable tissue segmentation option in InForm. Cell segmentation within tumor and stroma regions was performed using the parameters including minimum nuclear size and splitting sensitivity and the signals of the nuclei, cytoplasm, and membrane components as individual cells. Once the machine learning cell segmentation algorithm was optimised, cells were then phenotyped. CD34+, CD68+, CD56+, CD66b+, FOXP3+, CD8+, CD14+, CD3+, CD45+, CD20+ and CD4+ cells from MSIs were counted. Finally, the phenotype counts, density, and mean expression data were analysed using phenoptrReports (Akoya Biosciences) in R to generate the data for cell densities/area (mm^2^).

### Hematoxylin & Eosin (H & E) staining for serial sections of YTMA-76

To count total TILs in the same TMA sections where molecular subtypes of cells were determined, coverslips were removed, and H & E staining was performed. The fluorescently stained YTMA-76 serial sections were incubated overnight with gentle shaking in 10X TBST to remove the coverslips. The YTMA-76 sections were stained with Hematoxylin (Dako) for 5 minutes, followed by Eosin Y for 60 seconds. The brightfield H & E images were digitised at 20X using the ScanScope AT2 platform (Leica Biosystems, Wetzlar, Germany).

### Statistical analysis

Statistical analyses were performed using GraphPad Prism 9.1.0 (GraphPad Software Inc., CA, USA) and R. studio 1.4.1106 (Inc., Boston, MA). The cut-points for cell types and area variables were determined using X-tile cut-point finder.^20^ Kaplan–Meier plots for disease-specific survival (DSS) and overall survival (OS) were computed and comparisons were made by the log-rank test using survival and survminer packages in R studio. *Post hoc* Benjamini-Hochberg (BH) multiple comparisons test was performed when the results for each variable in survival analyses were significant. ROC curves were constructed from logistic regression models for the prediction of DSS. All statistical tests were two-sided, and significance was represented as (*) *p* < 0.05, (**) *p* < 0.01, (***) *p* < 0.001, (****) *p* < 0.0001, or not significant (ns). To perform univariable analyses of each factor, a Cox proportional hazards model was fitted to predict survival from each factor in turn. For the multivariable analysis, a Cox proportional hazards model was learned using eTIL%, age, gender and stage as predictors. These variables were chosen, since they were all present in a common group of 3 cohorts. For each test, we quote the hazards ratio associated with each level of a factor compared to the base reference level, and the associated p-values.

### Role of funding source

None of the funders were directly involved in the study design, data collection, analyses, interpretation, or writing of the manuscript. The corresponding author (David L Rimm) has full access to all the data and the final responsibility for the decision to submit for publication.

## Results

### Measurement of eTIL% as a prognostic variable in cohorts from multiple-institutions

**Figure 1:**
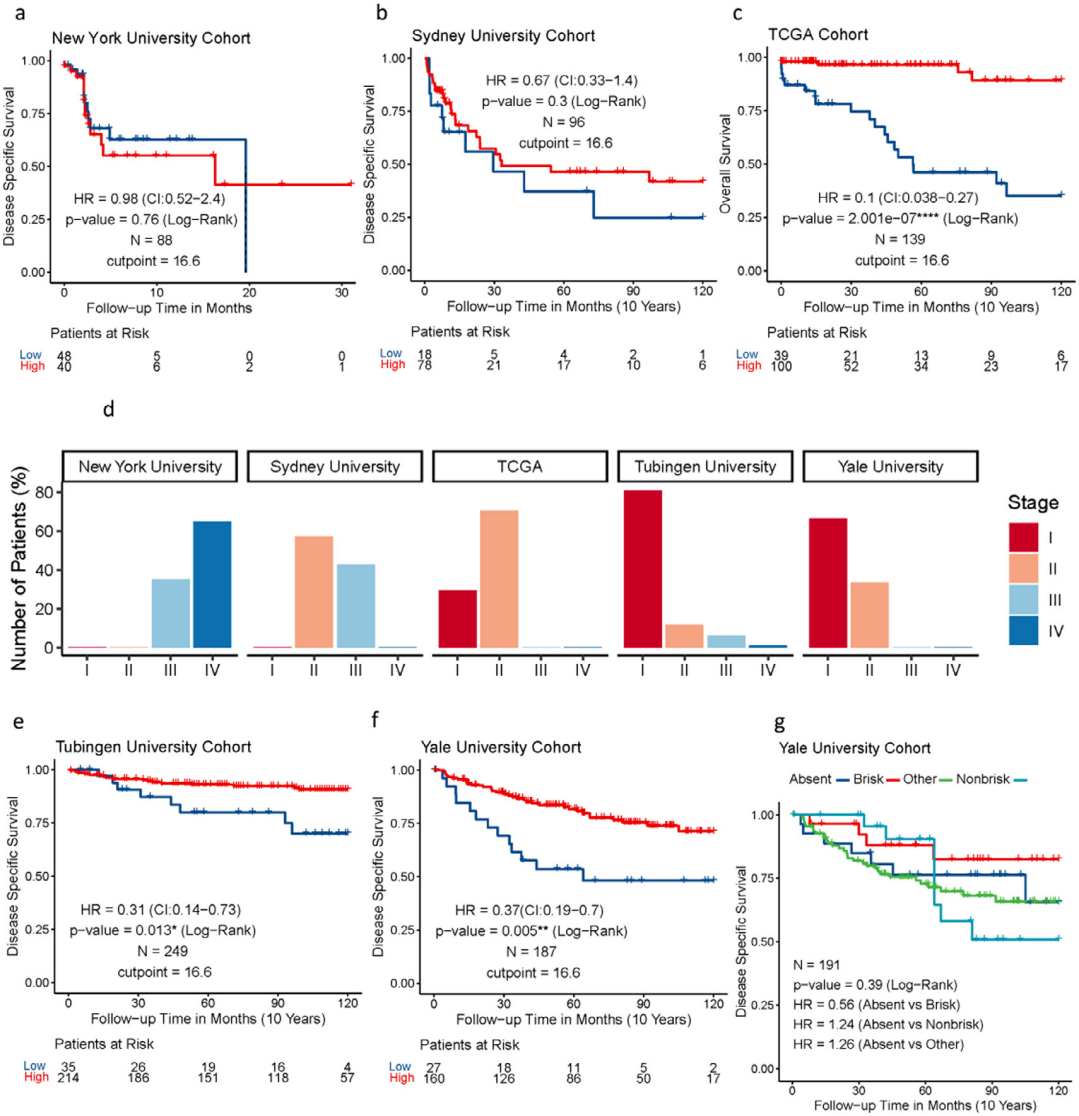
Assessment of eTIL% using the NN192 machine learning cell classifier and the established cut-point of 16.6% in the five cohorts from multiple institutions. (**a**) Kaplan-Meier curves of DSS in WTS NYU validation cohort (**b**) DSS in WTS Sydney University cohort, (**c**) OS in WTS TCGA cohort, (**e**) DSS in TMA Tubingen University Cohort, (**f**) DSS in TMA Yale University Cohort by eTIL% dichotomised at 16.6 % (G) pathologist's TIL scores. (**d**) Bar plot depicting the stage distribution of each cohort.

**Figure 2:**
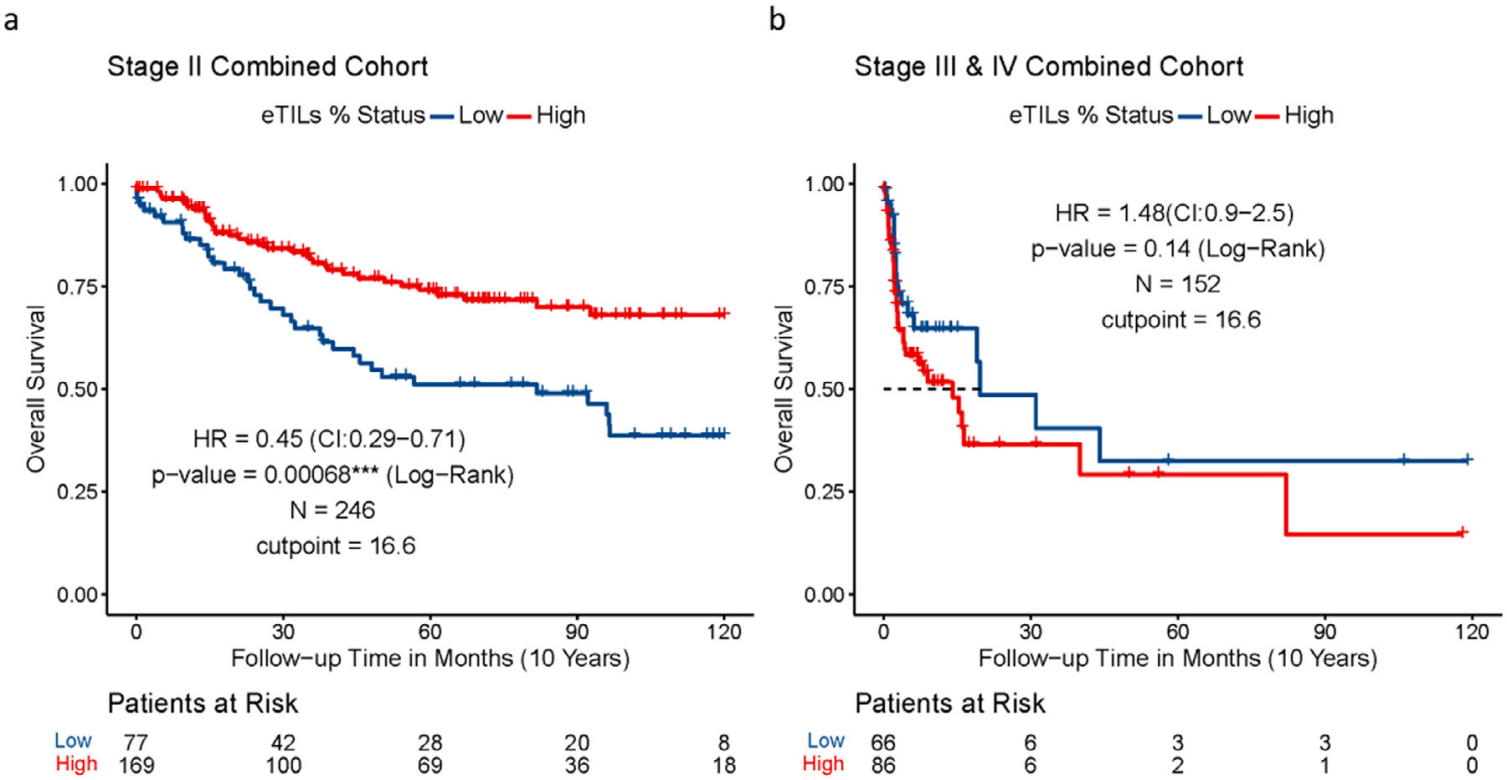
Assessment of eTIL% in stage specific combined cohorts. (**a**) Kaplan-Meier curve of OS in stage II only combined cohort and (**b**) Kaplan–Meier curve of OS in stage III and IV combined cohorts by eTIL% dichotomised at 16.6 %.

Assessment of eTIL% using the NN192 machine learning cell classifier and the established cut-point of 16.6 % in the five cohorts from multiple institutions showed that high eTIL% was associated with longer overall survival in TCGA cohort (hazard ratio (HR) = 0.1, *p* < 0.0001), better disease-specific survival in Tubingen cohort (HR = 0.31, *p* = 0.013), and Yale cohort (HR = 0.37, *p* = 0.005) (Figure 1c, 1e, 1f), but not in the NYU or Sydney cohorts (Figure 1a, 1b). However, the assessment of visual pathologist-read TILs in Yale Cohort showed no significant association with disease specific survival (Log-Rank *p* = 0.39, Figure 1g). Evaluation of the stage distribution of these cohorts showed that approximately 98% of patients in the NYU cohort and 43 % of patients in the Sydney cohort were stage III and IV patients, whereas stage III and IV patients in the Tubingen, Yale and TCGA cohorts were 6%, 0% and 0% respectively (Figure 1d). The results indicated that assessing eTIL% scores as a prognostic factor for patients with melanoma may be stage dependent as high eTIL% were associated with better prognosis mainly in patients with stage I and II disease. We generated a combined cohort containing 764 patients from Yale, Tubingen, Sydney, TCGA, and NYU cohorts. Univariable and multivariable analyses were performed to assess the association of eTIL% and the clinical pathological features with survival (Table 2). eTIL% (with a predefined 16.6 % cut-point), Stage, Breslow, ulceration and histogenesis were all significantly associated with survival in univariable analyses. The multivariable analysis showed that eTIL% was a significant predictor, even when combined with the other pathological features in a single model. Further, in an analysis of stage 2 only patients of the combined cohort with the 16.6 % cut-point, the results showed that higher eTIL% is associated with better prognosis (HR = 0.45, *p* = 0.00068) (Figure 2a), but not stage III and IV (HR = 1.48, *p* = 0.14) (Figure 2b). Our results could support the previous finding that patients with eTIL% >=16.6 have a significantly better prognosis.

**Table 2:** Univariable and multivariable Cox-proportional Hazards Regression analyses to assess the association of eTIL% and the clinical pathological features regarding disease-specific overall survival in all stage multi-institutional combined cohort.

Variables	Univariable Analysis		Multivariable Analysis	
	HR (95% CI)	*P* value	HR (95% CI)	*P* value
eTILs%	0.4 (0.29-0.55)	***<0.001***	0.5 (0.30-0.75)	***0.001***
Age	1.0 (0.99-1)	0.63	1.0 (0.98-1.01)	0.751
Gender (M vs F)	0.7 (0.46-1.1)	0.11	0.7 (0.46-1.09)	0.116
Stage (vs I)				
II	3.0 (2.1-4.3)	***0.001***	1.5 (0.87-2.41)	0.153
III	5.3 (3.4-8.1)	***0.001***	3.1 (0.98-9.91)	0.055
IV	19.9 (10.2-38.8)	***<0.001***	6.0 (2.56-14.12)	***<0.001***
Breslow (vs <0.8)				
1>4.0	8.1 (4.5-14.6)	***<0.001***		
10.8-1.0	3.2 (1.5-6.7)	***0.002***		
11.0-2.0	2.4 (1.4-4.4)	***0.003***		
12.0-4.0	4.3 (2.4-7.7)	***<0.001***		
Ulceration (Yes vs No)	2.0 (1.2-3.2)	***0.01***		
Histogenesis (11 Categories)				
	Max: 2.90E+07 (NA)	***<0.001***		
	Min: 9.97E-01 (NA)

### Assessment of five TIL variables in multi-institutional stage I and II combined cohort

**Figure 3:**
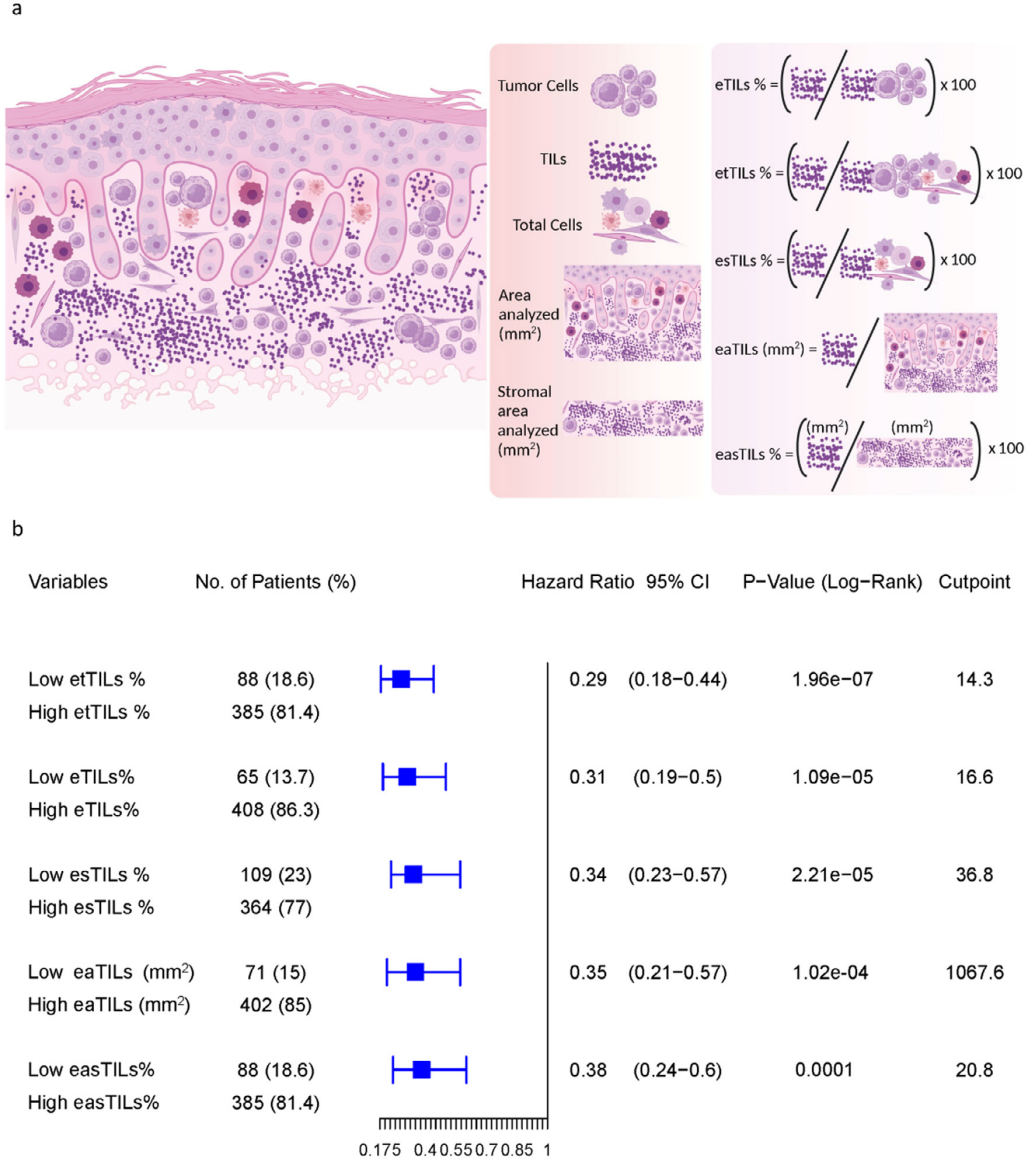
Assessment of five TILs variables including eTILs, etTILs, esTILs, eaTILs and easTILs. (**a**) Schematic diagram illustrating the variables (created with BioRender.com). (**b**) Forest plot of DSS in stage I and II combined discovery set. The optimal cut-points defined in the TCGA cohort as a training set for each variable, the *p*-values (log-rank) and HRs with 95% CI derived for measurement of the cohorts from assessing the optimal cut-points were shown.

To identify the best approach to measure eTILs for potential future clinical adoption, we tested five different methods to assess the densities and proportions of eTILs based on the cell types and the area analyzed. We used the TCGA cohort as a training set to find every possible cut-point and the association of each TIL variable with patient outcome. The optimal cut-points defined in the TCGA cohort for each variable, the p-values, and HRs derived for measurement of the cohorts from assessing the optimal cut-points are shown. Since the purpose of this assessment was to compare the performance of five variables, it is statistically unsound to compare the p-values of each variable but ok to compare the HRs to determine relative prognostic strength. We compared the estimated difference between the HRs, with confidence intervals (CIs), between variables, and reported the p-value evaluating the null hypothesis of no association between the prognostic variable and outcome. Our results show a significant association of all variables with OS (Figure 3b). The HRs between all five TIL variables are similar, but eTILs (HR=0.31, 95% CI (confidence interval) =0.19-0.5, *p* < 0.0001) and etTILs (HR=0.29, 95% CI=0.18-0.44, *p* < 0.0001) appeared to be more robust methods in the combined cohort (although not statistically significantly better). This indicates that eTILs and etTILs might be better performing methods than the remaining three methods in large future cohorts. This is concordant with the prognostic results with eTIL% previously reported in melanoma.^1^

### Identification of molecular subtypes of TILs

**Figure 4:**
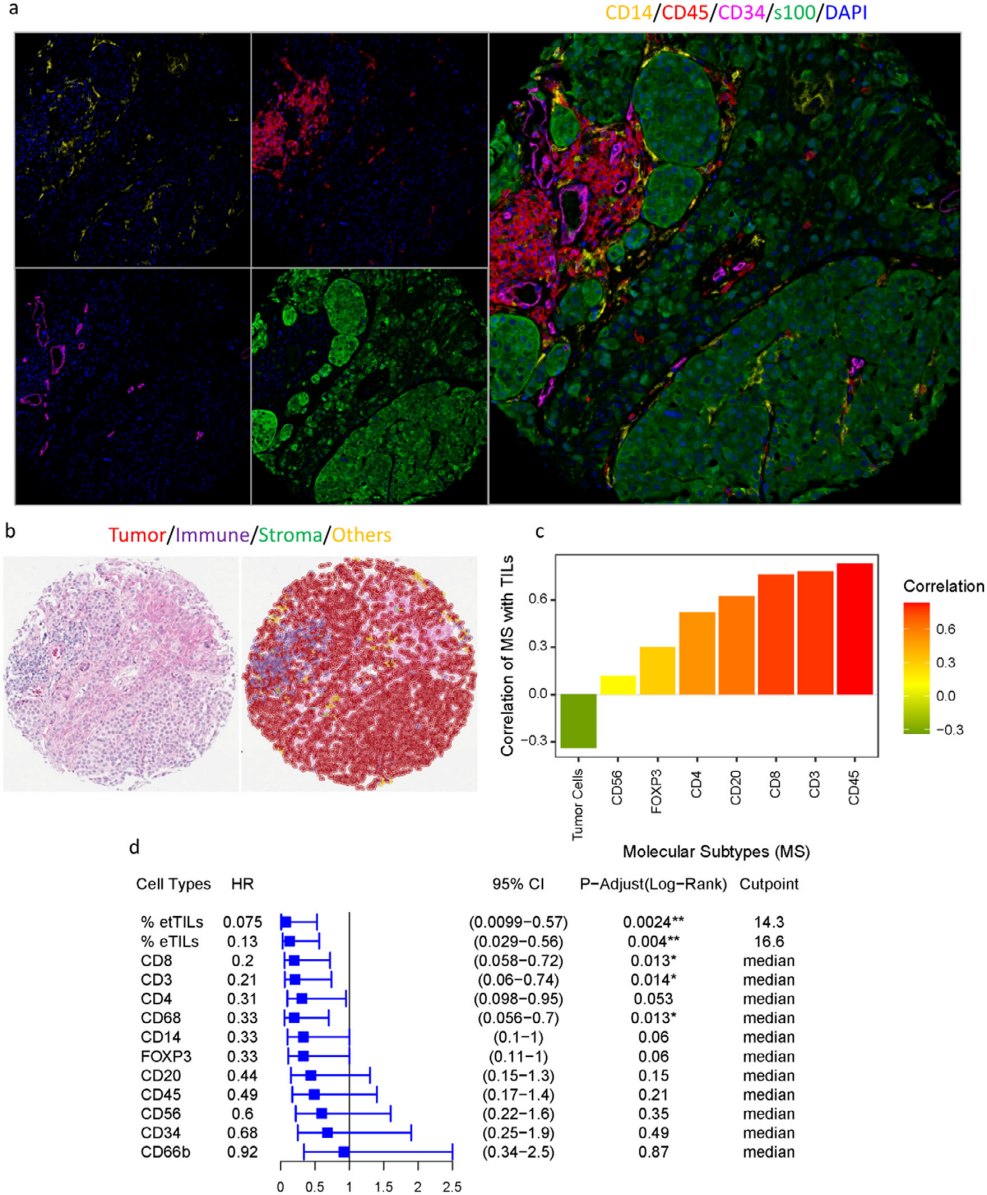
Identification of molecular subtypes of TILs. (**a**) Representative multispectral IF images of the panel (CD14/CD45/CD34) profiling in YTMA-76, tissue block 2, cut-64. (**b**) The representative H & E image and cell classification on the same tissue section using NN192. (**c**) Pearson linear regression between each molecular subtype of cells and TILs. (**d**) Forest plot of DSS in stage YTMA-76. The predefined cut-point 16.6% for eTILs, the optimal cut-point 14.3 defined in the TCGA cohort for etTILs and the median cut-points for all other cell types were used to stratify the patients. The adjusted p-values (log-rank, adjusted by Benjamini and Hochberg, BH) and HRs with 95% CI derived for measurement of the cohorts from assessing the optimal cut-points were shown.

Lymphocytic subtypes of TILs were identified by multiplexed IF on the serial sections of a Yale melanoma cohort (YTMA-76). Representative multispectral IF (MIF) images of the first panel (CD14/CD45/CD34) were shown in Figure 4a and of the panel (CD4/CD8/FOXP3/S100B) were shown in Supplementary Figure 4. The representative image of H & E staining on the same tissue section and cell classification using NN192 is shown in Figure 4b. In addition to the lymphoid lineage markers including CD3, CD4, CD8, CD20, CD45, CD56, and FOXP3, we also examined myeloid lineage markers such as CD14, CD68, CD34 to accurately identify the specific cell types within the tumor microenvironment of YTMA-76. The relationship between each molecular subtype of cell and TILs% was assessed by Pearson linear regression which showed a high positive correlation of TILs with cell types including CD45 (R = 0.83, *p* < 2.2e-16), CD3 (R = 0.78, *p* = 8.6e-15), CD8 (R = 0.76, *p* = 8e-14), CD20 (R = 0.62, *p* = 1.4e-08), CD4 (R = 0.52, *p* = 6.8e-06) and a weak correlation with cell types such as FOXP3 (R = 0.3), CD56 (R = 0.12) and CD66b (R = 0.02) (Figure 4c). These results indicate that the lymphocytic phenotypic marker most highly correlated to TILs is CD45 (leukocyte common antigen), and the specific phenotypic subtype of cells representing TILs may be CD3+ or CD8+ or CD4+ T cells. This was similarly reported previously in melanoma.^1^^,^^21^ These findings were corroborated by the cell type-specific survival analyses, which showed similar profiles (Supplementary Figure 1). The Kaplan–Meier estimates of survival test using the median as a cut-point showed that patients with high cell counts of CD3 (HR = 0.21, *p* = 0.015) and CD8 (HR = 0.2, *p* = 0.015), as well as myelocytic macrophage marker CD68 (HR = 0.33, *p* = 0.015) have significantly improved DSS (Figure 4d). As accumulated evidence showed that CD3+ and CD8+ lymphocytic cell infiltration is the primary determinant of immunotherapy outcome, evaluating the combination of both TILs and a group of specific molecular subtypes of TILs in association with the clinical outcome might assist in defining a subset of patients that might respond to immunotherapy.

**Figure 5:**
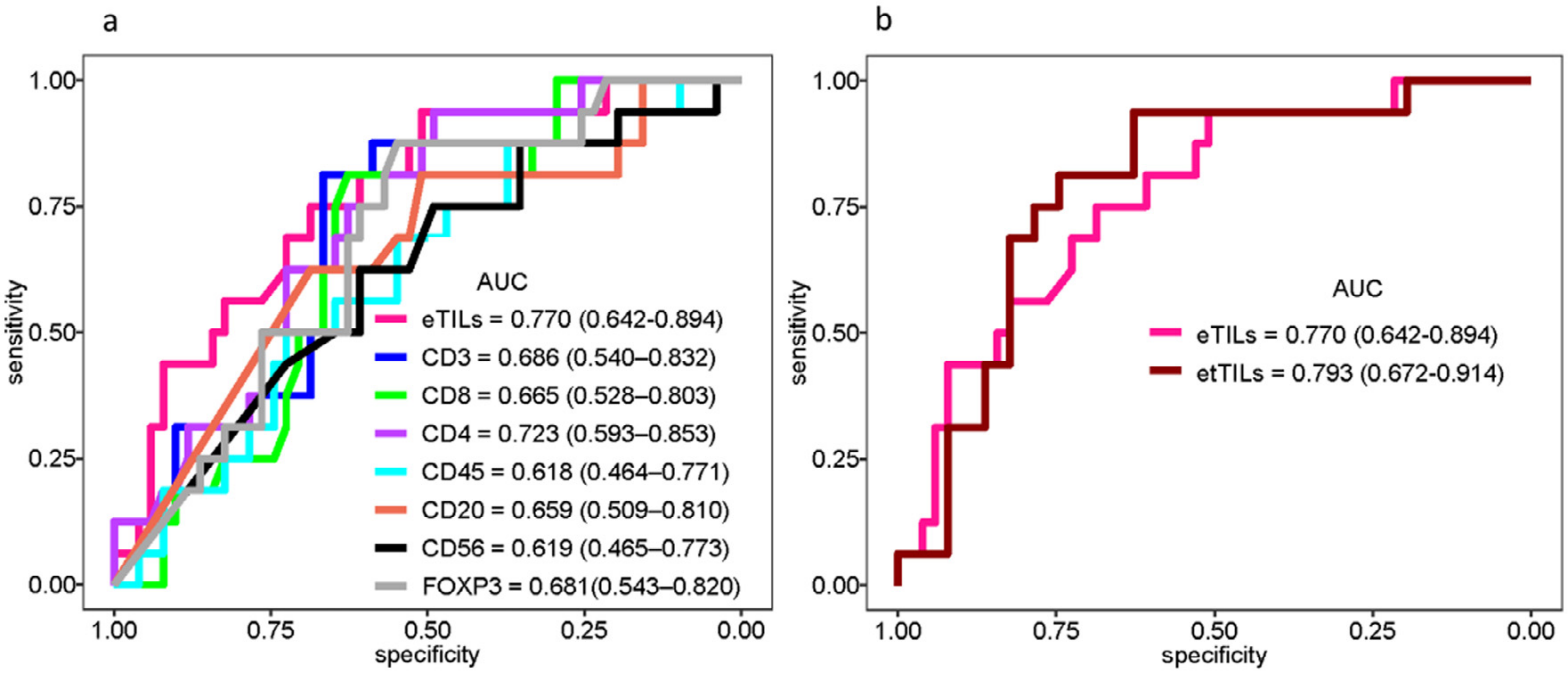
Identification of TIL variables for use as clinical utilities. (**a**) ROC curves indicating predictive accuracy of the markers: eTILs, CD3, CD8, CD4, CD45, CD20, CD56 and FOXP3. (**b**) ROC curves showing predictive accuracy, sensitivity, and specificity of eTILs and etTILs variables.

Next, we assessed the utility of TILs and specific molecular subtypes of cells to predict event risk. We generated the area under the receiver operating characteristic (ROC) curves (C-statistic) for TILs variables such as eTILs and etTILs, and all lymphocytic markers including CD4, CD3, CD8, CD45, CD20, FOXP3 and CD56 (Figure 5) to measure the risk prediction associated with these markers. eTILs achieved a favorable prognostic performance where the area under the curve (AUC) value for DSS was 0.77 (CI: 0.642-0.894). Of seven markers tested for AUC, CD4 (AUC: 0.723, CI: 0.593–0.853) showed the highest prognostic performance (Figure 5a). In agreement with the prior work,^21^ our results suggested that the prognostic value of TILs appeared to be driven by CD4+ T cells. The AUC of myelocytic macrophage marker CD68 was 0.678 (CI: 0.535-0.823), exhibiting a higher prognostic value. Our AUC analysis of etTILs variable predicting event risk in YTMA-76 showed that etTILs (AUC: 0.793, CI: 0.672-0.914, specificity: 0.627 and sensitivity: 0.938) outperformed eTILs (AUC: 0.77, CI: 0.642-0.897, specificity: 0.51 and sensitivity: 0.938) which validated our finding in a low-stage combined cohort from multiple institutions (Figure 5b). Taken together, assessment of ROC curves by comparing the clinical impact of two variable models (i.e., eTILs and etTILs) suggests that etTIL% might be a more robust variable to use clinically.

## Discussion

The use of immunotherapies, including Pembrolizumab and Ipilimumab in the adjuvant setting, has been shown to successfully manage stage III melanoma.^5^^,^^7^ However, since more patients with stage I or II melanoma are diagnosed than stage III,^22^ it is important to evaluate the role of these agents as adjuvant therapies in patients with early-stage melanoma. This is especially important because only one in five of stage III melanoma patients benefit from immunotherapy, and 50% are cured by surgery alone as seen in the placebo arm.^5^ These data suggest that we could identify the subset of patients that could potentially be spared immunotherapy toxicity since they are unlikely to benefit.^1^ Our study pools patients with melanoma from international cohorts and supports the previous finding that eTIL% score is an independent prognostic marker in melanoma and shows that the effect is seen only in low stage patients, the population containing the stage 2 patients that receive adjuvant therapy.

A highly reproducible estimate of the TIL calculation is needed to use the machine learning TIL score in the clinic. Unlike the NN192 algorithm, which relies on cell detection and classification, there are many machine learning models that train with patches rather than with cell detection to generate a TIL map that characterises lymphocytic infiltrates in intra-tumoral, peri-tumoral, and adjacent stromal regions.^23^ To identify the best approach for calculating TILs, we considered using multiple variables based on both the cell types (tumor cells, immune cells, fibroblasts, or other cells) and the area of interest (tumor and adjacent 1,2 mm diameter stroma). The test of five TIL variables in the low stage multi-institutional combined cohort showed that all TIL variables had comparable prognostic value; but eTIL% and etTILs % had the best performance. We note that TILs can also be predictive for immunotherapy, but evaluation of these variables in the context of immunotherapy is beyond the scope of this work.

A key limitation of our work is that we have used a machine learning algorithm that is susceptible to cell assignment error during cell classification. Another limitation is that melanoma cells have uneven membrane patterns and highly irregular cell shapes. Since melanoma cells change their shape and can adopt the appearance of other cells to invade any tissue in the body,^24^ it is often difficult to accurately classify tumor cells resulting in misclassification of malignant melanoma cells as the fibroblasts or other cells. As etTIL% was calculated using total cells as the denominator, the likelihood of incorrect percentage automated TIL calculation due to assignment error might be reduced as opposed to eTIL% method, which used the sum of only immune and tumor cells as its denominator. As a result, etTIL% may have higher reproducibility than eTIL% in patients with melanoma. However, we found no statistically significant difference between our two-best model variables. Finally, the use of 0.6-mm diameter tissue cores TMAs is an additional limitation of this study. The advantage of large numbers of cases accessible by TMA is the trade-off between assessing the fraction of the tumor and maximizing the number of samples. As such, the evaluation of TILs in both TMA and WTA formats show significant association with survival. Further we note the the TILs quantification done here is to illustrate the heterogeneity of the immune infiltrate that is recognized as eTIL. We do not attempt to validation the prognostic value of molecularly defined TIL in this work.

TILs comprise a heterogeneous cell population including natural killer (NK) cells, B cells and various subsets of T cells with complex functional states (e.g., naive, effector, memory, and dysfunctional) and thus, the prognostic value of the automated TIL may arise from the unbiased combination of distinct molecular subtypes.^25^ Here, we evaluated various molecular subtypes of lymphocytes within the TILs population and their prognostic values assessed by DSS. Concordant with the study by Piras, *et al.*,^26^ our results show that high CD8+ and CD3+ cells were associated with favorable DSS though the hazard ratio was higher than that of eTIL% and etTIL% variables (0.2 versus 0.13 and 0.075). In the study of Acs, *et al*., only a weak-fair correlation was reported between eTILs and CD4 and CD8 expression.^1^ The inherently subjective nature of the user-supervised training process for cell segmentation of IF images using cell segmentation algorithm might be a major contributor to this variation. The cell count analyses using IF cell segmentation platforms can lead to inconsistent outcomes, especially if the assessed cohort is not statistically powered. The provisional solution might be to use a combined application of both IF and H & E that are sufficiently generic to be easily trainable while consistently achieving high sensitivity and specificity with validation.

In summary, we validated that eTIL% score is a robust prognostic marker in patients with early-stage melanoma and identified distinct TIL subpopulations that carry the prognostic value. Pending prospective validation, the use of the NN192 machine learning algorithm might evolve into a useful and easy-to-implement tool that will aid in risk stratification of patients with early-stage melanoma. In the future, the use of eTILs might be complemented with molecular subtyping of cells for more discriminating analyses. The use of a combined marker signature may be proven to be the best approach to define a subset of patients that will not benefit from immunotherapy or might develop significant toxicities.

## Contributors

Conception and design: T.N. Aung, D.L. Rimm, Development of methodology: T.N Aung, Y. Bai, B. Acs, D.L. Rimm, Acquisition of data (provided animals, acquired, and managed patients, provided facilities, etc.): T.N. Aung, J. Wilmott, S. Nourmohammadi, V. Yaghoobi, T. Sinnberg, K. Khosrotehrani, I. Osman, J.F. Thompson, R.A. Scolyer, D.L. Rimm, Analysis and interpretation of data (e.g., statistical analysis, biostatistics, computational analysis): T.N. Aung, V. Yaghoobi, S. Nourmohammadi, I. Vathiotis, A. Fernandez, M. Moutafi, N. Gavrielatou, S. Martinez-Morilla, Writing, review, and/or revision of the manuscript: T.N. Aung, S. Shafi, J. Wilmott, S. Nourmohammadi, I. Vathiotis, N. Gavrielatou, T. Sinnberg, K. Khosrotehrani, I. Osman, V. Yaghoobi, Y. Bai, B. Acs, A. Fernandez, S. Martinez-Morilla, M. Moutafi, T. Amara, K. Ikenberg, J.F. Thompson, R.A. Scolyer, D.L. Rimm, Administrative, technical, or material support (i.e., reporting or organizing data, constructing databases): T.N. Aung, J. Wilmott, V. Yaghoobi, T. Sinnberg, T. Amara, K Ikenberg, K. Khosrotehrani, I. Osman, J.F. Thompson, R.A. Scolyer, D.L. Rimm, Study supervision: T.N. Aung, D.L. Rimm. Data verification: All data generated in this study will be accessible from the date this work is published by contacting D.L. Rimm (david.rimm@yale.edu) or T.N. Aung (thazin.aung@yale.edu). All authors read and approved the final version of the manuscript.

## Data sharing statement

Raw images were submitted to https://www.ebi.ac.uk/bioimage-archive with the accession number (S-BIAD470).

## Declaration of interests

David L. Rimm has served as an advisor for Astra Zeneca, Agendia, Amgen, BMS, Cell Signaling Technology, Cepheid, Daiichi Sankyo, Genoptix/Novartis, GSK, Konica Minolta, Merck, NanoString, PAIGE.AI, Perkin Elmer, Roche, Sanofi, Ventana and Ultivue. Astra Zeneca, Cepheid, NavigateBP, NextCure, Nanostring, Lilly, and Ultivue fund research in David L. Rimm's lab. Ana Bosch has participated in Advisory Board meetings for Pfizer and Novartis and received a travel grant from Roche. Dr. Rimm is supported for efforts in melanoma by Navigate Biopharma and the Yale SPORE in Skin Cancer: P50 CA121974 (M. Bosenberg and H. Kluger, PIs).

Richard A Scolyer has received fees for professional services from Evaxion, Provectus Biopharmaceuticals Australia, Qbiotics, Novartis, Merck Sharp & Dohme, NeraCare, AMGEN Inc., Bristol-Myers Squibb, Myriad Genetics and GlaxoSmithKline. Richard A Scolyer is supported by an Australian National Health and Medical Research Council Practitioner Fellowship. Richard A Scolyer and John F Thompson are the recipients of an Australian National Health and Medical Research Council Program Grant (APP1093017). John F Thompson has received honoraria for advisory board participation from BMS Australia, MSD Australia, GSK and Provectus Inc, and travel and conference support from GSK, Provectus Inc and Novartis.

Tobias Sinnberg reports grants from Neracare GmbH, Novartis, SkylineDx Pierre Fabre, and Pascoe GmbH and personal fees or travel support from BMS, Novartis, CeCaVa outside the submitted work.

CTSA Grant Number TL1 TR001864 supporting Dr. Aileen Fernandez from the National Center for Advancing Translational Science (NCATS), a component of the National Institutes of Health (NIH).

Iman Osman was supported by NCI R01 and NCI Melanoma SPORE.

Kiarash Khosrosthranni was supported by Cancer Council QLD grant 1125237 and Cancer Council QLD grant ACCR-000095.

Teresa Amaral was supported by Novartis, Neracare, Sanofi, SkylineDx and receives consulting fees from Pierre Fabre and BMS as an invited speaker. She acts as an advisor for CeCaVa.

Balazs Acs was supported for a postdoctoral grant supporting by The Swedish Society for Medical Research (Svenska Sällskapet för Medicinsk Forsknings - SSMF).

Saeed Nourmohammadi is supported for doctoral degree program by the University of Adelaide Research Scholarship.

All other authors report no conflicts of interest.

## Appendix Supplementary materials

Image, application 1
