# Supplementary material for: Objective assessment of tumor infiltrating lymphocytes as a prognostic marker in melanoma using machine learning algorithms
Source: eBioMedicine. 2022 Jul 7;82:104143. doi: 10.1016/j.ebiom.2022.104143 (PMC9272337; doi:10.1016/j.ebiom.2022.104143)
Supplement: Supplementary file 1 [file mmc1.docx]

**Supplementary Figure 1**. The cell type specific survival outcome analyses.

**Supplementary Figure 2**. Workflow for stain vector normalisation in QuPath. (a) Area section for stain vector estimation for individual image. The representative area was selected. (b) Estimate stain vector using “Auto” function. (c) Eosin stain vector before color normalisation and (d) after normalisation. (e) Hematoxylin stain vector before normalisation and (f) after normalisation.

**Supplementary Figure 3**. The number of cells identified as tumor and immune cells (in %) across multiple centers/cohorts.

**Supplementary Figure 4**. Representative multispectral IF images CD4/CD8/FOXP3/S100B panel.
